# Construction of an SNP fingerprinting database and population genetic analysis of 329 cauliflower cultivars

**DOI:** 10.1186/s12870-022-03920-2

**Published:** 2022-11-10

**Authors:** Yuyao Yang, Mingjie Lyu, Jun Liu, Jianjin Wu, Qian Wang, Tianyu Xie, Haichao Li, Rui Chen, Deling Sun, Yingxia Yang, Xingwei Yao

**Affiliations:** 1 Tianjin Academy of Agricultural Sciences, Tianjin, 300192 China; 2 College of Life Sciences, Nankai University, Tianjin, 300071 China; 3 National Key Facility for Crop Resources and Genetic Improvement, Institute of Crop Sciences, Chinese Academy of Agricultural Sciences, Beijing, 100081 China; 4 Tianjin Agricultural Development Service Center, Tianjin, 300061 China

**Keywords:** Cauliflower, SNP, KASP, DNA fingerprinting, Population structure

## Abstract

Cauliflower is one of the most important vegetable crops grown worldwide. However, the lack of genetic diversity information and efficient molecular markers hinders efforts to improve cauliflower. This study aims to construct DNA fingerprints for 329 cauliflower cultivars based on SNP markers and the KASP system. After rigorous filtering, a total of 1662 candidate SNPs were obtained from nearly 17.9 million SNP loci. The mean values of PIC, MAF, heterozygosity and gene diversity of these SNPs were 0.389, 0.419, 0.075, and 0.506, respectively. We developed a program for in silico simulations on 153 core germplasm samples to generate ideal SNP marker sets from the candidates. Finally, 41 highly polymorphic KASP markers were selected and applied to identify 329 cauliflower cultivars, mainly collected from the public market. Furthermore, based on the KASP genotyping data, we performed phylogenetic analysis and population structure analysis of the 329 cultivars. As a result, these cultivars could be classified into three major clusters, and the classification patterns were significantly related to their curd solidity and geographical origin. Finally, fingerprints of the 329 cultivars and 2D barcodes with the genetic information of each sample were generated. The fingerprinting database developed in this study provides a practical tool for identifying the authenticity and purity of cauliflower seeds and valuable genetic information about the current cauliflower cultivars.

**Supplementary Information:**

The online version contains supplementary material available at 10.1186/s12870-022-03920-2.

## Background

Cauliflower (*Brassica oleracea var. botrytis*), as an important vegetable crop grown worldwide, is gaining popularity in human diets because of its good flavor, rich nutritional value and anticarcinogenic effects [1]. Cauliflower production (including broccoli) has increased in recent decades, reaching 36.9 million tons in 2019 [2]. Since the late 1980s, China has become the world’s largest cultivation and production country of cauliflower [3]. The shared progenitor of *B. oleracea* species was believed to be *B. cretica* which originated in the eastern Mediterranean region [4, 5]. Cauliflower most likely evolved from broccoli and underwent a robust genetic bottleneck during its differentiation and domestication [6]. To date, reduced genetic variation and limited breeding resources have seriously slowed down the process of cauliflower improvement.

Over the past two decades, a series of DNA markers, including restriction fragment length polymorphism (RFLP) [7], random amplified polymorphic DNA (RAPD) [8], amplified fragment length polymorphism (AFLP) [9], inter-simple sequence repeat (ISSR) [10], and simple sequence repeat (SSR) [11] have been developed and widely used in the research fields of genetic diversity analysis, gene mapping, association studies, and molecular-assisted breeding, etc. In recent years, with the rapid development of high-throughput sequencing technologies, single nucleotide polymorphism (SNP) markers have gradually become the most popular option because of their relatively low cost, high yield, good stability and reproducibility, uniform distribution across the genome, and ease of documentation [12–17]. Until now, several PCR-based platforms, such as GoldenGate [18], Illumina Infinium [19], TaqMan from Life Technologies [20], and Kompetitive allele-specific PCR (KASP, LGC Bioresearch technologies), have been successfully developed for verification of SNP markers. Of these, the KASP platform, which takes advantages of high accuracy, high throughput, and cost-effectiveness, has been widely used in wheat [21–23], rice [24, 25], cotton [26], cucumber [27], and broccoli [28].

DNA fingerprinting is a powerful approach based on molecular markers or special sequences to identify genetic diversity and distinguish different plant cultivars [29, 30]. So far, DNA fingerprinting databases have been established in a series of plants such as maize [29, 31], grapevine [32], cigar tobacco [33], and red bayberry [34], demonstrating their technical significance for variety identification and germplasm innovation. In cabbage, 50 core SNPs were selected from 59 varieties and used in efficient identification of seed authenticity and purity [35]. In broccoli, deep sequencing was carried out for 23 representative broccoli lines, identifying 100 SNPs for subsequent KASP experiments and 25 core markers for fingerprinting. Further analyses of genetic diversity, genetic relationships, and population structure of 392 broccoli accessions revealed a narrow genetic background in the broccoli population [28]. In cauliflower, AFLP markers have been reported to explore the genetic diversity of different cauliflower varieties. The kinships between cauliflower and other subspecies of *B. oleracea* were revealed [36]. SSR markers were also widely used to reveal the genetic diversity and relationships of cauliflower varieties [37, 38]. However, so far, no SNP-based fingerprinting database has been reported in cauliflower.

Owing to the narrow genetic background and imperfect conservation and protection system, it is inconvenient and difficult for breeders to recognize cauliflower effectively and protect the variety rights [28]. Under these circumstances, an accurate, efficient, and economical method to support breeding efforts and protect plant variety rights is urgently needed for cauliflower. With SNP fingerprints for cauliflower, breeders could easily distinguish varieties using the high-throughput KASP detection platform instead of the traditional electrophoresis. Moreover, people can recognize variety information by comparing their genotyping results with this fingerprinting database of cauliflower cultivars constructed in our study.

This study developed a practical workflow to generate the SNP-based fingerprinting database for cauliflower. First, we screened high-quality SNP markers from the variation database previously generated by whole-genome resequencing. We then developed programs to select optimal marker combinations, followed by KASP conversion and fingerprinting of 329 representative cauliflower cultivars mainly collected from the public market. We also performed population genetic analysis and variety identification of these 329 cultivars based on the KASP genotyping results. Our results will provide practical tools for variety authenticity and purity identification and give a new sight of the genetic relationships of current commercial cauliflower cultivars.

## Results

### Screening of SNP markers

**Fig. 1:**
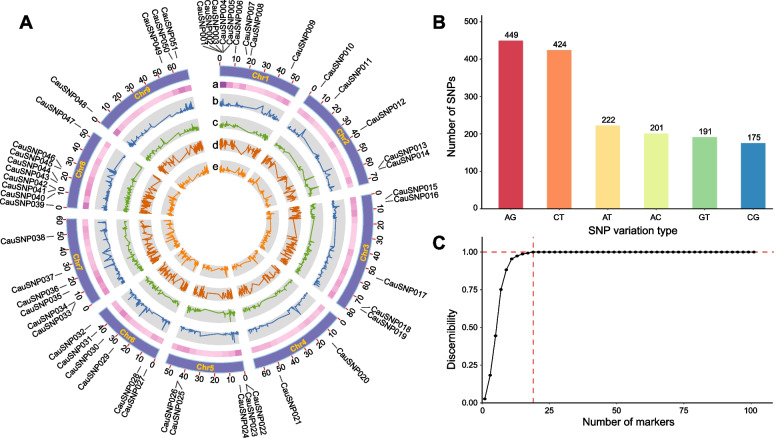
SNP screening and in silico simulation. **A** Distribution and statistics of the 1662 SNPs. Tracks toward the center: a, marker density; b, polymorphism information content (PIC); c, heterozygosity rate; d, minor allele frequency (MAF); e, gene diversity. The 51 successfully designed KASP markers were labeled. **B** Statistics of the six variant types of the 1662 SNPs. **C** In silico simulation of SNP marker discernibility in 153 core germplasm samples

According to the filtering criteria, including locus features, heterozygosity, missing rate, MAF, and the adjacent distance from each other, 1662 high-quality SNPs were identified and considered as a candidate pool for further selection, and were evenly distributed across the cauliflower genome except for the centromere regions (Fig. 1A). The statistics of the single base variations showed that A/G (27.02%) and C/T (25.51%) transitions were dominant types, followed by four types of transversions: A/T (13.36%), A/C (12.10%). G/T (11.50%) and C/G (10.53%). The ratio of transitions to transversions was approximately 1.11 (Fig. 1B), which was consistent with the previous study on cigar tobacco [33]. We then calculated the PIC, MAF, heterozygosity and gene diversity to assess the utility of the entire set of candidate SNPs. PIC and MAF values of these 1662 SNPs ranged from 0.363 to 0.504 and 0.343 to 0.475, with average values of 0.389 and 0.419, respectively. The majority of PIC values (80.75%) fluctuated between 0.38 to 0.50. Heterozygosity had a mean value of 0.075, ranging from 0.029 to 0.193. The mean value of gene diversity was 0.506, ranging from 0.473 to 0.591 (Fig. 1A, Table S2). The results of these indicators suggest that the 1662 candidate SNPs have a high degree of polymorphism and are ideal for DNA fingerprinting of cauliflower.

### In silico simulation and selection of optimal combinations of SNPs

We performed in silico simulations to systematically evaluate the discernibility of candidate SNPs for DNA fingerprinting development. Consequently, one combination of 19 SNPs could achieve complete identification of 153 accessions (Fig. 1C). There were 29 and 106 sets of 25 and 30 SNPs, respectively, that could also achieve perfect recognition of 153 cauliflower inbred lines. Considering the failure rate of primer design, we initially selected redundant SNP markers initially for KASP validation, which contained a total of 60 SNP loci derived from three optimal combinations of 20 SNPs.

### Conversion and genotyping of KASP markers

**Fig. 2:**
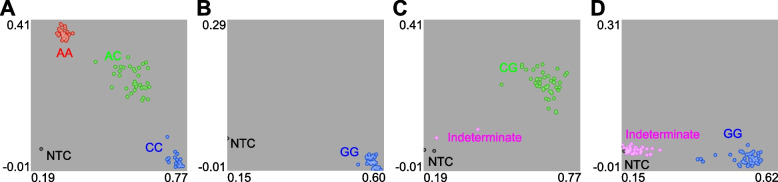
Representative results of fluorescence-labeled KASP markers. **A** A KASP marker with good polymorphism. **B** A KASP marker with homozygous monomorphism that should be discarded. **C** A KASP marker with heterozygous monomorphism that should be discarded. **D** A KASP marker with monomorphism and a high missing rate that should be discarded

Due to the lack of sequence specificity or abnormal GC content, 9 SNP markers could not be converted to KASP markers. With a conversion rate of 85%, 51 evenly distributed SNPs were successfully transformed into KASP markers and subjected to subsequent experiments (Fig. 1A, Table S3). KASP markers were first verified in 96 out of 329 cauliflower cultivars. According to the genotyping results, 2 markers failed to be amplified, and 8 markers that showed monomorphism or high missing rates of more than 10% were discarded for further analysis (Fig. 2). Finally, 41 high-quality KASP markers were retained and regarded as the core set of KASP markers (Table 1).

**Table 1:** Statistics of 41 core markers

Marker	Chromosome	Position	Ref	Alt	PIC	MAF	Gene Diversity	Heterozygosity
CauSNP001	Chr1	860,653	C	G	0.198	0.128	0.223	0.030
CauSNP002	Chr1	975,765	C	T	0.359	0.375	0.469	0.386
CauSNP003	Chr1	1,581,568	C	A	0.351	0.350	0.455	0.316
CauSNP004	Chr1	2,022,083	A	T	0.372	0.442	0.493	0.568
CauSNP005	Chr1	2,022,902	C	T	0.357	0.366	0.464	0.733
CauSNP006	Chr1	9,494,597	G	T	0.348	0.340	0.449	0.450
CauSNP007	Chr1	16,591,554	A	C	0.304	0.249	0.374	0.359
CauSNP009	Chr1	45,449,565	T	A	0.367	0.410	0.484	0.401
CauSNP010	Chr2	3,174,530	A	G	0.375	0.497	0.500	0.532
CauSNP011	Chr2	14,297,682	G	A	0.374	0.474	0.499	0.541
CauSNP012	Chr2	39,230,790	T	A	0.365	0.401	0.481	0.486
CauSNP013	Chr2	65,005,201	C	T	0.375	0.485	0.500	0.416
CauSNP014	Chr2	66,045,832	A	T	0.349	0.344	0.451	0.389
CauSNP016	Chr3	6,005,226	T	C	0.372	0.447	0.494	0.407
CauSNP018	Chr3	73,929,470	T	C	0.220	0.147	0.251	0.155
CauSNP019	Chr3	75,221,240	C	T	0.262	0.192	0.310	0.128
CauSNP020	Chr4	14,063,327	C	G	0.374	0.465	0.498	0.499
CauSNP021	Chr4	52,102,326	G	A	0.365	0.403	0.481	0.410
CauSNP022	Chr5	311,091	C	T	0.364	0.397	0.479	0.441
CauSNP023	Chr5	326,763	G	C	0.363	0.392	0.477	0.420
CauSNP024	Chr5	4,246,754	G	C	0.345	0.331	0.443	0.334
CauSNP025	Chr5	42,917,099	G	C	0.372	0.448	0.495	0.568
CauSNP026	Chr5	42,919,228	T	C	0.371	0.438	0.492	0.565
CauSNP027	Chr6	1,872,141	G	A	0.373	0.454	0.496	0.538
CauSNP030	Chr6	34,494,441	G	C	0.364	0.398	0.479	0.468
CauSNP031	Chr6	40,582,776	G	T	0.372	0.448	0.495	0.562
CauSNP032	Chr6	46,214,109	C	T	0.371	0.436	0.492	0.410
CauSNP033	Chr7	6,266,029	G	A	0.373	0.459	0.497	0.492
CauSNP034	Chr7	7,108,476	T	G	0.288	0.225	0.349	0.006
CauSNP036	Chr7	18,611,643	C	G	0.372	0.445	0.494	0.021
CauSNP037	Chr7	26,326,101	G	A	0.274	0.207	0.328	0.000
CauSNP038	Chr7	49,710,841	C	T	0.353	0.356	0.458	0.383
CauSNP039	Chr8	685,258	A	G	0.373	0.451	0.495	0.362
CauSNP040	Chr8	7,993,815	A	T	0.371	0.439	0.493	0.410
CauSNP041	Chr8	10,333,745	C	G	0.373	0.459	0.497	0.517
CauSNP042	Chr8	10,995,531	G	A	0.374	0.473	0.499	0.362
CauSNP044	Chr8	16,285,895	A	G	0.365	0.400	0.480	0.325
CauSNP045	Chr8	23,662,826	C	A	0.365	0.400	0.480	0.198
CauSNP046	Chr8	24,753,546	C	A	0.334	0.306	0.424	0.465
CauSNP047	Chr8	51,980,792	T	C	0.370	0.427	0.489	0.489
CauSNP048	Chr9	2,367,375	C	T	0.373	0.457	0.496	0.477

**Fig. 3:**
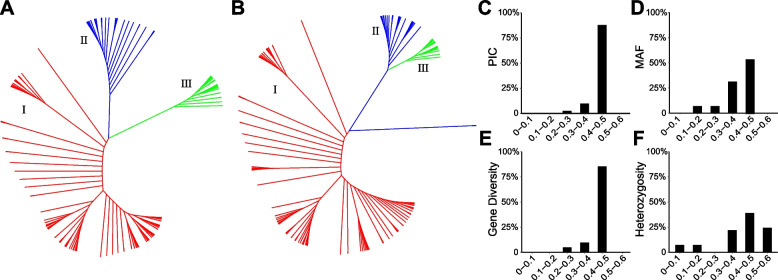
Genetic information content of 41 core markers. **A** Phylogenetic tree of 153 core germplasm samples constructed using 1662 SNPs. **B** Phylogenetic tree of 153 core germplasm samples constructed using 42 core SNPs. **C**-**F** Genetic information content of 41 core markers in 329 cauliflower cultivars including PIC, MAF, gene diversity, and heterozygosity rate, respectively

To further evaluate the discriminative power of the 41 SNP loci, we constructed phylogenetic trees of 153 core germplasm resources using these 41 SNPs and the entire 1662 SNPs, respectively. As shown in Fig. 3A and B, two phylogenetic trees showed very similar and consistent patterns, indicating that the core set of KASP markers exhibited a high level of polymorphism and could reflect the genetic diversity of cauliflower. In addition, we evaluated the performance of the 41 markers in 329 cauliflower accessions, which were mainly hybrid varieties collected from the public market and derived from domestic and foreign seed companies. As a result, most PIC, MAF and gene diversity values ranged from 0.3 to 0.5, indicating high genetic polymorphism. Notably, heterozygosity values of most KASP markers (85%) were more significant than 0.3 due to the high proportion of hybrids in the 329 cauliflower cultivars (Fig. 3C-F, Table 1).

### Phylogenetic analyses of 329 cauliflower varieties

**Fig. 4:**
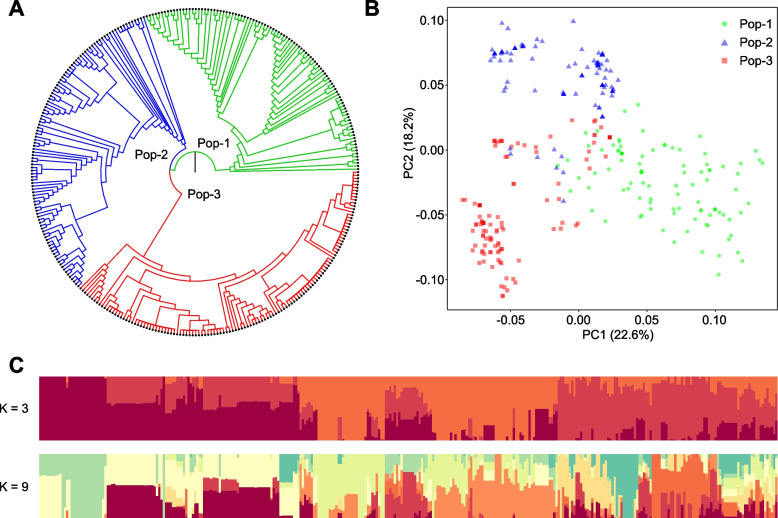
Phylogenetic and population genetic analyses of 329 cauliflower cultivars. **A** Phylogenetic tree of 329 cultivars constructed with 41 KASP markers. **B** Principal component analysis. **C** Population structure analysis of 329 cultivars at *K* = 3 and *K* = 9

We constructed a phylogenetic tree based on the genotyping data of 41 KASP markers for 329 cultivars using FastTree software. As shown in Fig. 4A, the cauliflower population could be clustered into three major groups, comprising 98 (Pop-1, green), 107 (Pop-2, blue) and 124 (Pop-3, red), respectively. A few subgroups were observed within the main groups, and some accessions were not fully distinguished, indicating the close genetic relationships among these cultivars. We further performed principal component analysis (PCA) based on the genotyping data, and the results were consistent with the phylogenetic tree. The first three principal components explained 22.6% (PC1), 18.2% (PC2), and 7.6% (PC3) of the total genetic variance, respectively (Fig. 4B). In contrast to Pop-1 and Pop-2, Pop-3 contained more aggregated points, reflecting the different degree of genetic diversity in the subgroups. In addition, the overlapping points in the central region among the three groups indicated possible genetic exchanges.

Population structure analysis was also performed with several groups (*K*) ranging from 1 to 15. Although the CV error rate reached the lowest value at *K* = 9, a classification of 3 groups could be accepted for all samples at *K* = 3. When *K* = 3, three kinds of components corresponding to the main groups could be clearly distinguished. The mixed colors in each group indicated that they were mutually connected and penetrated (Fig. 4C). In addition, the population structure results were confirmed by the clustering results of nucleotide identity analysis (Fig. S2). These results demonstrated that the 41 KASP markers could recognize most cultivars in the current seed market and were suitable for developing DNA fingerprints of cauliflower.

### Establishment and application of the DNA fingerprint

**Fig. 5:**
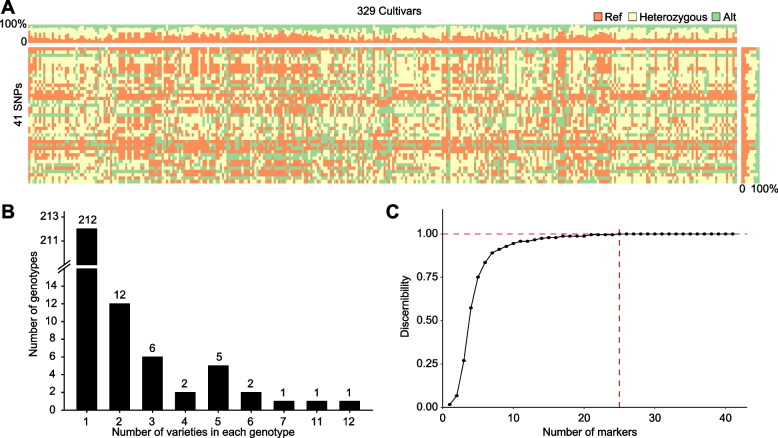
Fingerprinting of 329 cauliflower cultivars and its application. **A** DNA fingerprints of 329 cauliflower cultivars. Each line represents one SNP locus, and each column represents one accession. Orange, yellow and green represent 0/0, 0/1, and 1/1, respectively. **B** Genotypes identified by the 41 core markers. **C** Cumulative KASP marker efficiency compared with the 41 core markers for the 329 cultivars

To develop a rapid and cost-effective approach for cauliflower cultivar identification, we constructed a fingerprinting database by integrating the core set of 41 KASP markers and 329 cauliflower cultivars collected from the seed market (Fig. 5A). The uniform distribution of heterozygous and homozygous SNP sites confirmed the genetic diversity of these 41 KASP markers. A total of 242 different genotypes were produced and 212 accessions could be distinguished entirely from other cultivars. In comparison, the remaining 117 accessions had 30 identical genotypes (Fig. 5B). Interestingly, we even found 11 and 12 accessions, respectively, that shared two genotypes. These results may indicate the narrow genetic background of cauliflower possibly due to the use of similar elite plants as parents.

To further evaluate the marker efficiency and reduce the number of core markers, we modified the simulation program to generate 10,000 combinations at each number. As a result, at least 25 SNP markers could achieve the same discernibility as the total 41 KASP markers (Fig. 5C). Therefore, we confirmed two candidate core sets of 25 SNP markers that could be used for cauliflower variety identification to reduce experimental costs (Table S4). The online software Caoliaoerweima (http://cli.im/) was used to encode the genotyping data of the 41 core SNPs for 329 cauliflower cultivars, and a 2D barcode fingerprint was generated for each cultivar (Fig. S3).

## Discussion

Cauliflower is an important vegetable crop grown with high nutritional value in its edible curd. As a subspecies of *Brassica oleracea*, cauliflower experienced a robust genetic bottleneck during its domestication, resulting in a narrow genetic background severely limiting its improvement and breeding [6]. To facilitate the utilization of germplasm and the protection of variety rights, it is necessary to understand the genetic relationships and population genetic structure among varieties/cultivars at the genomic level. SNP has become the ideal marker for genetic analysis due to its stability and high-throughput reproducibility. Moreover, SNP markers can be easily verified using KASP technology [29]. SNP fingerprinting based on KASP markers has proven to be a reliable, accurate and cost-effective approach for variety identification and protection in many crops and horticultural plants such as wheat [21], rice [25], maize [29], cabbage [35], and broccoli [28], etc. In cauliflower, traditional markers such as AFLP [36] and SSR [37, 38] have been used for genetic analysis and fingerprinting inbred and hybrid lines of cauliflower. However, no SNP-based fingerprinting system has been established for cauliflower, making it urgently necessary to develop an efficient and accurate fingerprinting platform for variety authenticity and purity identification and population genetic analysis. In this study, we obtained 1662 high-quality SNPs from the variation database generated by whole-genome resequencing. We further performed in silico simulations to select optimal marker sets for DNA fingerprinting. Finally, we constructed an SNP-based fingerprinting database with 41 core KASP markers and comprehensively analyzed the genetic relationships and population structure of 329 commercial cauliflower cultivars.

To obtain high-quality and polymorphic SNPs, we set up a stringent pipeline for filtering. Notably, only SNPs at fourfold degenerate sites (4D-SNPs) were retrieved because they were under weak directional selection and exhibited more extensive polymorphism [39]. As a result, 1662 SNPs were screened from 17.9 million SNP sites, which significantly increased the efficiency of the subsequent selection of core markers. For a further selection of desirable SNP loci for KASP marker design, we developed an in silico algorithm to evaluate the efficiency of different combinations of SNP loci instead of relying on descriptive statistics and artificial selection. Consequently, 60 SNPs were selected, of which 51 were successfully transformed into KASP markers with a conversion rate of 85%. After excluding 10 low-quality SNP loci, the remaining 41 KASP markers formed the core marker set. The bioinformatic filtration approach and the in silico simulation followed by KASP verification could serve as an excellent example for designing and optimizing the process of DNA fingerprint development.

Most of the 329 cauliflower cultivars were collected from the public seed market. Fingerprinting hybrid lines from the market has more practical value than previous studies focusing mainly on inbred germplasm [28,38]. Therefore, heterozygosity values were higher than in previous studies and ranged from 0 to 0.733 with an average of 0.391. Owing to the biallelic nature of the SNP, the PIC value of SNPs is limited to 0 to 0.5 [40]. The mean PIC-value of 0.380 in this study is considered moderate compared to the value of 0.43 of SSR marker [38] and 0.33 in broccoli [28]. In addition, MAF values ranged from 0.128 to 0.479 with an average of 0.384. The MAF threshold significantly affects fingerprinting and infers population structure [41]. SNPs with low MAF values tend to be less polymorphic than those with higher MAF values. 85.4% of KASP markers had a gene diversity value between 0.4 and 0.5 with a mean value of 0.456, suggesting the practicality of the core markers used in the present study. In conclusion, the 41 SNP loci selected in this study are qualified and suitable for fingerprinting and population analysis.

Population structure within the cauliflower subspecies remains obscure because of the substantial bottleneck during domestication [6]. According to the results of phylogeny and principal component analysis, 329 cauliflower cultivars were divided into 3 main groups. About 81% (79) of the cultivars in Pop-1 were compact-curd cauliflowers. Pop-2 consisted of 107 varieties, of which 86% were compact-curd type imported from abroad. Most of the 124 cultivars in Pop-3 were loose-curd cauliflowers, mainly from the provinces of China such as Shanghai, Zhejiang, Fujian, and Taiwan. In previous studies, Cai et al. classified the cauliflower populations into winter, summer/autumn and tropical types [6]; Zhu et al. used 43 SSR markers to analyze the genetic diversity of 165 cauliflower inbred lines and divided the accessions into 4 categories, whereas the clustering patterns did not match traits such as curd maturity, curd solidity or geographic origin [38]. Rakshita et al. divided 96 genotypes of Indian cauliflower and related crops into 4 clusters with a composite pattern of genotype distribution [42]. Our results indicate that the discernibility of the core marker set is reliable, and the classification patterns of most studied cauliflower cultivars essentially corresponded to the geographic origin and degree of curd solidity. In addition, using hybrid lines could promote the practical application value of DNA fingerprinting in the seed market and plant variety protection.

As a subspecies of *Brassica oleracea*, cauliflower has a narrow genetic background and undergoes a short breeding history in China. In this study, we stringently selected 41 SNP markers for fingerprinting 329 cauliflower cultivars, which can distinguish most of the representative commercial cultivars in the current seed market. Theoretically, we pressumed that at least 25 core KASP markers were sufficient to identify the cauliflower cultivars, despite some samples sharing identical genotypes due to the close genetic relationship or possible synonyms. The DNA fingerprinting database developed in this study will contribute to the protection of plant breeders’ rights, the utilization of germplasm resources and the genetic improvement of cauliflower. Meanwhile, there is still room for perfecting the current fingerprinting system by elevating the accuracy and efficiency of variety identification. In the near future, with the advances in breeding technology and the increase in the number of varieties, it may also be necessary to integrate more specific SNP markers such as those closely associated with the important agronomic traits of cauliflower.

## Conclusions

In this work, we integrated whole-genome resequencing data and KASP technology to detect SNP loci and generate a fingerprinting database for a population of 329 cauliflower cultivars collected from the public market. 41 SNPs formed the core marker set and were used to construct SNP fingerprints of 329 cauliflower germplasm resources. The 329 cauliflower cultivars could be well divided into 3 clusters, of which the classification pattern was consistent with the geographic origin and degree of curd solidity. Our results have demonstrated the reliability and preciseness of SNP markers and the practical value of DNA fingerprinting technology. They will be able to fill the gap in identifying the varietal authenticity and purity of cauliflower in the current commercial market.

## Materials and methods

### Plant materials

A total of 329 cauliflower accessions were collected from the public market and stored at the Tianjin Kernel Vegetable Research Institute, Tianjin Academy of Agricultural Sciences (Table S1). This collection has a diverse genetic background and abundant phenotypic variation that could represent the majority of cauliflower cultivars worldwide including China, Japan, Europe and America. All cultivars were planted in 2021 in the field of Wuqing experimental base, Tianjin, China. Young leaves were sampled at the seedling stage for genomic DNA extraction.

### DNA extraction

The sample leaves were pestled with a 4 mm steel ball in a Retsch MM 400 Mixer Mill after chilling in liquid nitrogen. The genomic DNA extraction was performed using a modified CTAB-Phenol–chloroform method, as described previously [43]. DNA concentration and purity were detected by using a NanoDrop™ 2000 spectrophotometer (Thermo Fisher Scientific, USA). The DNA with a concentration ≥ 25 ng/μL and normal optical density (OD) (260/230 ≥ 1, 1.5 ≥ 260/280 ≤ 2.2) was used for subsequent KASP genotyping experiment.

### SNP marker screen and statistics

In our laboratory, 820 cauliflower inbred lines were subjected to whole-genome resequencing on NovaSeq 6000 Sequencing System (Illumina, USA) with a 150 bp paired-end sequencing strategy. After removing the sequencing adaptors and low-quality reads with fastp software [44], the clean reads were mapped to the modified reference genome cauliflower inbred line C-8 [45] using BWA software (v.0.7.17) with default options [46]. The modified genome sequence data has been deposited in the Genome Warehouse in National Genomics Data Center, Beijing Institute of Genomics, Chinese Academy of Sciences / China National Center for Bioinformation, under accession number GWHBJSH00000000 that is publicly accessible at https://ngdc.cncb.ac.cn/gwh. Then, duplicate reads were removed using the GATK (v.4.0.10.0) MarkDuplicates function [47]. GATK HaplotypeCaller was used to generate raw variants (SNPs and InDels). The raw SNPs were filtered using GATK VariantFiltration with parameters “QD < 2.0, MQ < 40.0, FS > 60.0, MQRankSum < -12.5, ReadPosRankSum < -8.0, --cluster-size 3 --cluster-window-size 10”, and a DNA variation database containing 17.9 million SNPs for cauliflower was generated. Based on the variation database, we developed a pipeline including SNP filtration, in silico simulation, KASP marker design, fingerprint construction and population genetic analysis (Fig. S1). We performed the filtration for selecting candidate SNPs from the whole SNP loci according to the following criteria: (1) biallelic sites; (2) 4-fold degenerate SNPs; (3) heterozygosity rate < 0.2; (4) missing rate < 0.05; (5) minor allele frequency (MAF) > 0.3; (6) no InDels or SNPs in the 100 bp flanking region. The information of 1662 filtered SNP loci used in this study has been uploaded to Zenodo with accession number 7179139 (https://zenodo.org/record/7179139). BCFtools (v.1.9) and VCFtools (v.0.1.13) were used for downstream filtering [48]. Genetic diversity parameters such as polymorphic information content (PIC), heterozygosity rate, MAF and gene diversity (expected heterozygosity) were calculated using PowerMarker v3.25 software [49].

### In silico simulation of core SNPs selection

To obtain the optimal set of SNPs for cauliflower cultivar identification, we developed a Python-based program to select the most suitable set of SNPs by in silico simulation on 153 core germplasm samples of cauliflower (https://github.com/Lvmingjie/SNP-fingerprints-of-Cauliflower, CoreSNPSimulation.py). The program would randomly select a specific number of SNPs (1 to 100 in this study) for 5000 times, generating 5000 combinations of SNPs. Then, all marker information (A, C, G, or T) of each line (Table S5) was extracted and joined to a string type. Finally, the strings which represented the characterization of samples were compared and the number of unique strings were calculated. The discernibility of a marker set was calculated using the following formula:

$$discernibility=\frac{unique\ string\ number}{total\ sample\ number}$$

Only the set of SNPs which could distinguish the maximum number of cauliflower cultivars were selected as candidate SNP loci.

### Design of KASP markers and genotyping

The upstream and downstream 100 bp sequences around the candidate SNP loci were extracted for KASP marker design. These flanking sequences were aligned to the genome with BLASTN for removing non-specific sequences. Unique sequences were selected for subsequent analysis. For each target site of KASP, two allele-specific and one common primer were designed. Primer design parameters were set as follows: (1) GC content < 60%; (2) melting temperature (Tm) between 55 and 62 °C; (3) PCR product size not larger than 120 bp. The Sangon Biotech Co., Ltd. (Shanghai, China) synthesized the primers and FAM- or VIC-tail. The KASP primers were designed by using the Primer Premier software V6.10 (https://www.premierbiosoft.com/primerdesign/index.html) according to the previous study [22]. All primers used in this study are listed in Table S3. Individual samples were amplified in 5 μl reactions in 384-plate as described previously [50]. Fluorescence detection of the reactions was performed using a BMG POLARstar Omega scanner, and data were analyzed using KlusterCaller 3.4.1 software. The detection data were visualized with the SNPviewer 2.0 software.

### Population structure and phylogenetic analyses

The experimental results of KASP markers for 329 samples were collected and transformed to VCF format using a customized Python script. Then the missing data were imputed with BEAGLE (v5.2) software [51]. Principal component analysis (PCA) was performed using GCTA (v1.940) software [52]. The phylogenetic tree was constructed using FastTree (v2.1.11) [53] and illustrated with FigTree (v1.4.4) (http://tree.bio.ed.ac.uk/software/figtree/). Model-based clustering results and population structure were analyzed with ADMIXTURE (v1.3.0) and R language [54]. The cross-validation (CV) error was calculated, and the *K* value corresponding to the lowest CV value was considered the optimal subpopulation results.

## Supplementary Information

**Additional file 1.**

**Additional file 2.**

**Additional file 3.**

**Additional file 4.**

## Data Availability

The datasets presented in this study can be found in online repositories. The names of the repository/repositories and accession number(s) can be found below: https://www.ncbi.nlm.nih.gov/bioproject/;PRJNA794342; https://zenodo.org/record/7179139.
