# Supplementary figures and images for: Construction of an SNP fingerprinting database and population genetic analysis of 329 cauliflower cultivars
Source: BMC Plant Biol. 2022 Nov 10;22:522. doi: 10.1186/s12870-022-03920-2 (PMC9647966; doi:10.1186/s12870-022-03920-2)

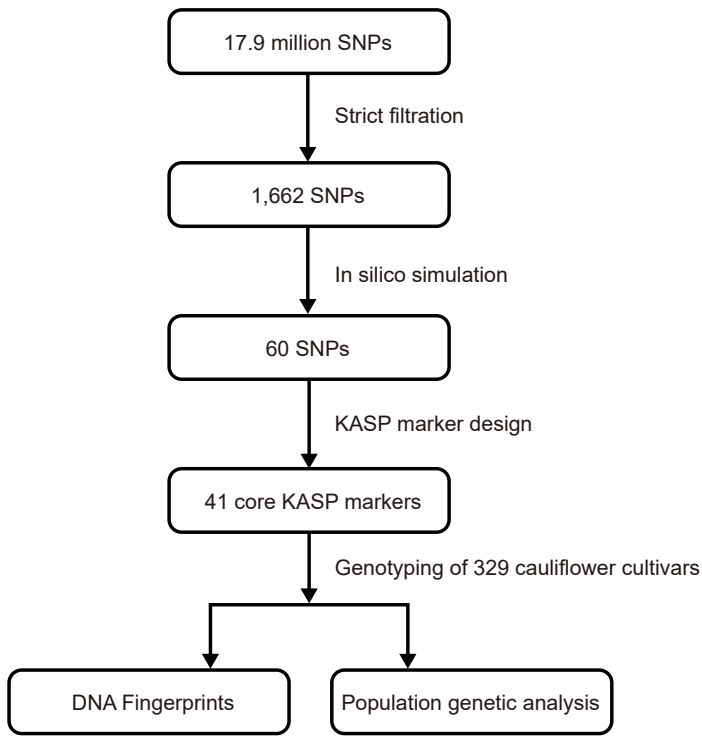

Fig. S1. Overview of the workflow of SNP-based fingerprinting of 329 cauliflower cultivars

Supplement: Supplementary file 2 — Additional file 2. [file 12870_2022_3920_MOESM2_ESM.pdf]

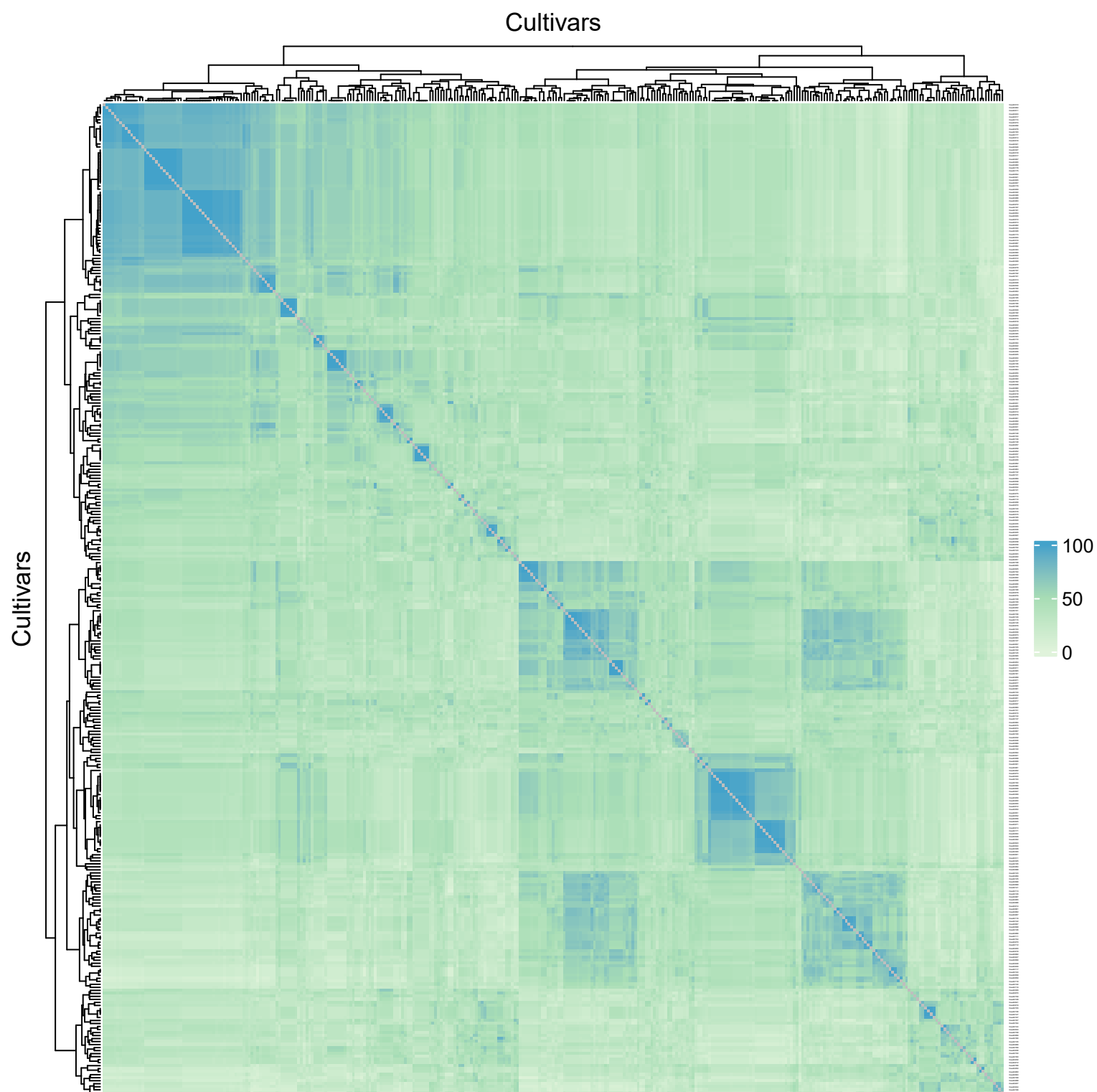

Fig. S2. Nucleotide identity matrix of the 329 cultivars.

Supplement: Supplementary file 3 — Additional file 3. [file 12870_2022_3920_MOESM3_ESM.pdf]
