# Supplementary material for: Construction of an SNP fingerprinting database and population genetic analysis of 329 cauliflower cultivars
Source: BMC Plant Biol. 2022 Nov 10;22:522. doi: 10.1186/s12870-022-03920-2 (PMC9647966; doi:10.1186/s12870-022-03920-2)
Supplement: Supplementary file 4 — Additional file 4. [file 12870_2022_3920_MOESM4_ESM.pdf]

CauAC001

Fig. S3. 2D barcodes for the 329 cultivars.

CauAC002

CauAC003

CauAC004

CauAC005

CauAC006

CauAC007

CauAC008

CauAC009

CauAC010

CauAC011

CauAC012

CauAC013

CauAC014

CauAC015

CauAC016

CauAC017

CauAC018

CauAC019

CauAC020

CauAC021

CauAC022

CauAC023

CauAC024

CauAC025

CauAC026

CauAC027

CauAC028

CauAC029

CauAC030

CauAC031

CauAC032

CauAC033

CauAC034

CauAC035

CauAC036

CauAC037

CauAC038

CauAC039

CauAC040

CauAC041

CauAC042

CauAC043

CauAC044

CauAC045

CauAC046

CauAC047

CauAC048

CauAC049

CauAC050

CauAC051

CauAC052

CauAC053

CauAC054

CauAC055

CauAC056

CauAC057

CauAC058

CauAC059

CauAC060

CauAC061

CauAC062

CauAC063

CauAC064

CauAC065

CauAC066

CauAC067

CauAC068

CauAC069

CauAC070

CauAC071

CauAC072

CauAC073

CauAC074

CauAC075

CauAC076

CauAC077

CauAC078

CauAC079

CauAC080

CauAC081

CauAC082

CauAC083

CauAC084

CauAC085

CauAC086

CauAC087

CauAC088

CauAC089

CauAC090

CauAC091

CauAC092

CauAC093

CauAC094

CauAC095

CauAC096

CauAC097

CauAC098

CauAC099

CauAC100

CauAC101

CauAC102

CauAC103

CauAC104

CauAC105

CauAC106

CauAC107

CauAC108

CauAC109

CauAC110

CauAC111

CauAC112

CauAC113

CauAC114

CauAC115

CauAC116

CauAC117

CauAC118

CauAC119

CauAC120

CauAC121

CauAC122

CauAC123

CauAC124

CauAC125

CauAC126

CauAC127

CauAC128

CauAC129

CauAC130

CauAC131

CauAC132

CauAC133

CauAC134

CauAC135

CauAC136

CauAC137

CauAC138

CauAC139

CauAC140

CauAC141

CauAC142

CauAC143

CauAC144

CauAC145

CauAC146

CauAC147

CauAC148

CauAC149

CauAC150

CauAC151

CauAC152

CauAC153

CauAC154

CauAC155

CauAC156

CauAC157

CauAC158

CauAC159

CauAC160

CauAC161

CauAC162

CauAC163

CauAC164

CauAC165

CauAC166

CauAC167

CauAC168

CauAC169

CauAC170

CauAC171

CauAC172

CauAC173

CauAC174

CauAC175

CauAC176

CauAC177

CauAC178

CauAC179

CauAC180

CauAC181

CauAC182

CauAC183

CauAC184

CauAC185

CauAC186

CauAC187

CauAC188

CauAC189

CauAC190

CauAC191

CauAC192

CauAC193

CauAC194

CauAC195

CauAC196

CauAC197

CauAC198

CauAC199

CauAC200

CauAC201

CauAC202

CauAC203

CauAC204

CauAC205

CauAC206

CauAC207

CauAC208

CauAC209

CauAC210

CauAC211

CauAC212

CauAC213

CauAC214

CauAC215

CauAC216

CauAC217

CauAC218

CauAC219

CauAC220

CauAC221

CauAC222

CauAC223

CauAC224

CauAC225

CauAC226

CauAC227

CauAC228

CauAC229

CauAC230

CauAC231

CauAC232

CauAC233

CauAC234

CauAC235

CauAC236

CauAC237

CauAC238

CauAC239

CauAC240

CauAC241

CauAC242

CauAC243

CauAC244

CauAC245

CauAC246

CauAC247

CauAC248

CauAC249

CauAC250

CauAC251

CauAC252

CauAC253

CauAC254

CauAC255

CauAC256

CauAC257

CauAC258

CauAC259

CauAC260

CauAC261

CauAC262

CauAC263

CauAC264

CauAC265

CauAC266

CauAC267

CauAC268

CauAC269

CauAC270

CauAC271

CauAC272

CauAC273

CauAC274

CauAC275

CauAC276

CauAC277

CauAC278

CauAC279

CauAC280

CauAC281

CauAC282

CauAC283

CauAC284

CauAC285

CauAC286

CauAC287

CauAC288

CauAC289

CauAC290

CauAC291

CauAC292

CauAC293

CauAC294

CauAC295

CauAC296

CauAC297

CauAC298

CauAC299

CauAC300

CauAC301

CauAC302

CauAC303

CauAC304

CauAC305

CauAC306

CauAC307

CauAC308

CauAC309

CauAC310

CauAC311

CauAC312

CauAC313

CauAC314

CauAC315

CauAC316

CauAC317

CauAC318

CauAC319

CauAC320

CauAC321

CauAC322

CauAC323

CauAC324

CauAC325

CauAC326

CauAC327

CauAC328

CauAC329
